# Diabetes mellitus and sensorineural hearing loss: is there an association? Baseline of the Brazilian Longitudinal Study of Adult Health (ELSA-Brasil)

**DOI:** 10.6061/clinics/2017(01)02

**Published:** 2017-01

**Authors:** Alessandra G Samelli, Itamar S Santos, Renata R Moreira, Camila M Rabelo, Laurie P Rolim, Isabela J Bensenõr, Paulo A Lotufo

**Affiliations:** I Departamento de Fisioterapia, Fonoaudiologia e Terapia Ocupacional, Faculdade de Medicina da Universidade de São Paulo, São Paulo/SP, Brazil; II Centro de Pesquisa Clínica e Epidemiológica, Hospital das Clínicas da Faculdade de Medicina da Universidade de São Paulo, São Paulo/SP, Brazil; III Departamento de Clínica Médica, Hospital das Clínicas da Faculdade de Medicina da Universidade de São Paulo, São Paulo/SP, Brazil; IV Serviço de Audiologia, Hospital das Clínicas da Faculdade de Medicina da Universidade de São Paulo, São Paulo/SP, Brazil

**Keywords:** Diabetes Mellitus Type II, Hearing Loss Sensorineural, Audiometry

## Abstract

**OBJECTIVES:**

Although several studies have investigated the effects of diabetes on hearing loss, the relationship between these two conditions remains unclear. Some studies have suggested that diabetes may cause sensorineural hearing loss, whereas others have failed to find an association. The biggest challenge in investigating the association between diabetes and hearing loss is the presence of confounding variables and the complexity of the auditory system. Our study investigated the association between diabetes and sensorineural hearing loss. We evaluated the influence of time from diabetes diagnosis on this association after controlling for age, gender, and hypertension diagnosis and excluding those subjects with exposure to noise.

**METHODS:**

This cross-sectional study evaluated 901 adult and elderly Brazilian Longitudinal Study of Adult Health (ELSA-Brasil) participants from São Paulo, Brazil who underwent audiometry testing as part of ELSA-Brasil’s baseline assessment.

**RESULTS:**

Hearing thresholds and speech test results were significantly worse in the group with diabetes than in the group without diabetes. However, no significant differences were found between participants with and without diabetes after adjusting for age, gender, and the presence of hypertension. Hearing thresholds were not affected by occupational noise exposure in the groups with and without diabetes. In addition, no association between the duration of diabetes and hearing thresholds was observed after adjusting for age, gender, and hypertension.

**CONCLUSION:**

We found no association between the duration of diabetes and worse hearing thresholds after models were adjusted for age, gender, and the presence of hypertension.

## INTRODUCTION

The burden of diabetes is increasing worldwide 1, including in Brazil 2–4. In 1986, a national survey reported a prevalence of diabetes for people aged 30–69 years of 7.6% 2, and by 2000, the prevalence had increased to 12.1% 3. Most recently, the elderly Brazilian Longitudinal Study of Adult Health (ELSA-Brasil) found a frequency of diabetes mellitus of 19.8% for adults aged 35–74 years 4.

In addition to the cardiovascular outcomes and the long-term damage to the kidneys, eyes, and nervous system caused by diabetes 5, this disease was also associated with alterations in hearing function in a meta-analysis of 18 clinical and epidemiological studies 6.

The pathological support to this association may be related to an increase in capillary lesions in the cochlea, more specifically in the stria vascularis and basilar membrane. Other studies have also reported a reduction in the number of spiral ganglion neurons. These differences could be related to the duration of diabetes and to comorbidities that could affect the inner ear 7.

Although several studies have investigated the effects of diabetes on hearing loss (HL), the relationship between these two conditions remains unclear. Some studies have suggested that diabetes may cause sensorineural hearing loss (SNHL) 6,8–10, whereas others have failed to find an association 11–15. The biggest difficulty in investigating the association between diabetes and HL is the presence of confounding variables and the complexity of the auditory system 16,17.

In this study, we investigated the association between diabetes and SNHL. We evaluated the influence of other factors such as age, gender, exposure to noise, hypertension diagnosis, and duration of diabetes on this association.

## MATERIALS AND METHODS

This study was approved by the Ethics Committee of the University Hospital at University of São Paulo (n 883/09).

### Study design

In this ancillary cross-sectional study, we evaluated 901 ELSA-Brasil participants from São Paulo (total ELSA-Brasil participants in São Paulo, 5,061) who were invited to participate in the study and agreed to undergo audiometry testing as part of ELSA-Brasil’s baseline assessment. Informed consent was given by all participants. The study was approved by the Ethics Committee of the University Hospital at University of São Paulo (n 883/09).

The design, objectives, and cohort profile of ELSA-Brasil have been published in detail elsewhere 18,19. The ELSA-Brasil is a prospective cohort study of 15,105 civil servants from six institutions in different Brazilian cities (São Paulo, Belo Horizonte, Porto Alegre, Salvador, Rio de Janeiro, and Vitória). All active or retired employees aged 35–74 years were eligible for the study. The baseline assessment consisted of a 7-hour examination, which took place from August 2008 to December 2010. Blood samples were taken after an overnight fast, and an oral 75 g glucose tolerance test and glycated hemoglobin measurements were performed 20.

### Hearing examination

After otological inspection, an audiological assessment was conducted. Screening acoustic immittance measurements (Madsen Otoflex 100) were performed to exclude middle ear disorders.

Pure-tone audiometry was performed using air conduction at octave frequencies from 250-8,000 Hz and bone conduction at 500-4,000 Hz.

Speech tests included the Speech Reception Threshold (SRT) and the Speech Discrimination Score (SDS). The SRT assesses people’s ability to hear and understand standardized three-syllable words (threshold in decibel hearing level (dBHL)), and the SDS evaluates people’s ability to hear and understand standardized one-syllable words (percentage of words correctly identified).

All tests were performed using a Madsen Itera II audiometer in a sound-proof room.

### Study variables

Socio-demographic characteristics and medical and occupational histories were obtained. Diabetes was defined as follows: medical history of diabetes; reported use of medications to treat diabetes; fasting serum glucose ≥7 mmol/l; glycated hemoglobin (HbA1c) level ≥48 mmol/mol; or 2-h oral glucose tolerance test with 75 g of glucose ≥11.1 mmol/l. Hypertension was defined as follows: reported use of medications to treat hypertension; systolic blood pressure ≥140 mmHg; or diastolic blood pressure ≥90 mmHg. Dyslipidemia was defined as follows: reported use of lipid-lowering treatment or low-density lipoprotein (LDL) cholesterol level ≥3.36 mmol/l.

Audiometric and speech test variables were compared between left and right ears in each group (D: group with diabetes and ND: group without diabetes). No significant differences were found between the left and right ears, they were grouped. Then, we calculated the mean values (both ears together) for the following measures: each audiometric frequency (threshold by frequency); low- to middle-range frequencies (250–2,000 Hz); high-range frequencies (3000–8,000 Hz); SRT and SDS. For each audiometric frequency mean, a value >25 dBHL was considered HL 21.

### Statistical analysis

Continuous variables were expressed as means ± SD, and categorical variables were expressed as proportions. The chi-square and Kruskal-Wallis tests and one-way analysis of variance (ANOVA) were used whenever applicable. We constructed linear regression models using the hearing threshold values, SRT, and SDS for each frequency as dependent variables to evaluate their association with diabetes. The following models were constructed: 1 crude, 2 adjusted for age; and 3 adjusted for age, gender, and hypertension diagnosis. We also built binary logistic regression models to study the association between hearing impairment in each frequency range (low-middle or high) and diabetes diagnosis. The odds ratio (OR) was calculated considering the number of individuals with hearing impairment in each frequency range (low-middle or high) in the D and ND groups.

We constructed similar models including only individuals with diabetes (N=191) to determine if the time from diabetes diagnosis was associated with HL. In this analysis, we set cutoff times at 1, 5, 7, and 9 years from diagnosis. We also performed a sensitivity analysis excluding individuals with a history of noise exposure. All analyses were performed using R software v.3.1.2. The significance level was set at 0.05.

## RESULTS

Of the 901 participants, 191 (21.2%) had diabetes. Among those with diabetes, HbA1c levels were available for 190 participants (99.5%), and adequate glycemic control (<53 mmol/mol) was found in 149 (78.4%) participants. Table 1 shows the baseline characteristics for the ELSA-Brasil study population. The mean time from diagnosis was 4.0±5.13 years, and the age at diagnosis ranged from 30 to 72 years (50.47±9.57 yrs).

Table 1 shows that participants in the D group were older and included more men and more participants with hypertension compared to the ND group. In addition, glucose, HbA1c, systolic and diastolic blood pressure, triglycerides, and creatinine levels were significantly higher in the D than the ND group. Results for audiometry testing at 250–8,000 Hz, SRT, and SDS were significantly worse in participants in the D than ND group.

Table 2 shows the number of participants with HL in each frequency range (low-middle and high). The number of participants with HL was significantly higher in the D group than the ND group. The OR for both frequency ranges showed a difference between the groups (crude model). However, in the adjusted model, this difference between groups was lost.

We also analyzed the audiometric measurements using linear models. Table 3 shows the beta coefficients for the association between audiometric measurements and a diagnosis of diabetes. In this analysis (crude model), all audiometric measurements and speech test variables were significantly worse in the D group than the ND group. However, the differences between groups were not significant when the models were adjusted for age. This lack of significance remained after further adjustment for gender and hypertension diagnosis.

We also performed a subgroup analysis of participants with diabetes to evaluate the association between hearing loss and time from diabetes diagnosis. Among the 191 participants, 3 (1.6%) could not precisely time their diabetes diagnosis and were excluded from these analyses. Among the remaining 188 individuals, 98 (52.1%) did not report themselves as diabetic and were diagnosed based on the results of the ELSA-Brasil laboratory baseline assessment. Therefore, these subjects were considered as having a time from diabetes diagnosis of zero. Participants in the D group were grouped according to the time from diagnosis (<9 years or ≥9 years). Although the mean hearing threshold for most frequencies and the SRT were lower in participants diagnosed ≥9 years than in those diagnosed <9 years, no significant differences were found after adjusting for age, gender, and the presence of hypertension (*p*-values 0.627; 0.489; 0.785; 0.611; 0.477; 0.914; 0.207; 0.277; 0.172; and 0.478, respectively). Similarly, no significant differences in hearing thresholds or speech tests were found between the D and ND groups at 1, 5, or 7 years from diagnosis after adjusting for age, gender, and presence of hypertension.

**Figure 1:**
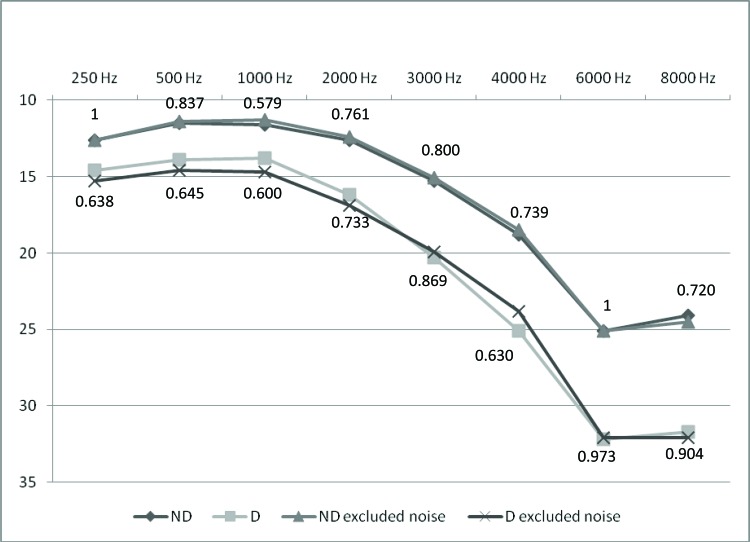
Means of hearing thresholds for ND (n=710) and D (n=191) groups (without exclusion of noise effect) and for ND excluded noise (n=450) and D excluded noise (n=120); *p*-values.

We conducted another analysis excluding the 71 participants in the D group (37%) and 260 participants in the ND group (36.6%) with a history of noise exposure. In both groups, the mean hearing thresholds were not significantly different after these participants were removed from the analysis. The *p*-values for the comparison of hearing thresholds between groups considering the whole sample or only those without a history of noise exposure are shown in Figure 1.

## DISCUSSION

We investigated the association between diabetes and SNHL in ELSA-Brasil participants from the São Paulo investigation center. In crude models, we found significant differences between the D and ND groups in most variables evaluated, including demographic characteristics (age and gender), fasting blood collection and blood pressure variables. Participants in the D group were older, and there were also more men and more hypertensive participants in the D than ND group.

Although the LDL and total cholesterol levels were higher in the D group, no significant differences were found between groups. Conversely, triglyceride levels were significantly higher in D than in ND participants. Similar findings were reported by Kakarlapudi et al. 22, who suggested that the similar cholesterol levels may be because persons with diabetes are more aggressively treated with antilipemic medications such as statins that lower LDL and total cholesterol but have a weaker effect on triglyceride levels.

In the crude model, pure-tone audiometry thresholds and speech test results were significantly worse in the D group than the ND group (Table 1). These findings are in agreement with studies showing that people with diabetes are at a high risk of HL 6,8,16,22–25.

Analysis of the crude model (Table 2) supports these previous findings 6,8,16,22–25 because the number of participants with HL was significantly higher in the D than ND group at both low- to middle-range frequencies and high-range frequencies. However, analysis of an adjusted model (Table 2) showed no difference between the D and ND groups at either low- to middle-range or high-range frequencies.

A meta-analysis by Horikawa et al. 9 included data from 13 studies (20,194 participants and 7,377 cases) that compared the prevalence of HL between D and ND adults. Type 1 and 2 diabetes participants (15–84 years old) with a duration of diabetes from 4 to >10 years were included (some studies did not provide this information). The overall pooled OR of HL for D compared to ND participants was 2.15 (95% CI: 1.72–2.68), and the prevalence of HL was higher in D participants than in ND participants across studies.

A meta-analysis by Akinpelu et al. 6 included data from 18 studies and obtained results (OR: 1.91; 95% CI: 1.47–2.49) similar to our crude analysis. This meta-analysis included people with type 2 diabetes (26–70 years old) and a disease duration of 2.9–14.6 years (some studies did not provide this information). Thus, the ORs reported by Horikawa et al. 9 and Akinpelu et al. 6 were similar to that obtained in this study (crude analysis), even though the participant characteristics differed across studies and the average age of the participants and the mean duration of diabetes were lower in our study.

Uchida et al. 16 argued that the association between diabetes and HL could not be strongly supported because many confounding variables may affect this association, including noise exposure and presbycusis. Indeed, other studies that investigated the effects of diabetes on hearing function did not remove confounding factors such as exposure to noise 22,26,27, gender 22,26,28, age 26, and hypertension 6,9,16.

Thus, we attempted to control for these possible confounding factors in our study and determine whether the differences observed in the initial analyses (Tables 1 and 2 – crude analysis) were also present (i.e., D participants had lower hearing thresholds than ND participants) after models were adjusted for age, gender, hypertension diagnosis, and exposure to noise.

Hearing threshold and speech test results were not significantly different between the D and ND groups after adjusting for age, gender, and presence of hypertension (Table 3), and the number of participants with HL was not significantly higher in the D than ND group at either low- to middle-range frequencies or high-range frequencies in the adjusted model (Table 2).

This last analysis suggested that age contributed most to the worse hearing thresholds of participants with diabetes observed previously (Tables 1 and 2 – crude model), which does not support the findings of other studies 9,16,29 showing a more severe effect of diabetes on hearing in younger age groups. Nevertheless, we cannot rule out residual confounding by age, especially in older age groups.

Regarding speech tests, few studies have compared results between subjects with and without diabetes 12,26. Concerning the results obtained in this study, before the adjustments for sex, age and hypertension, there was a difference between the D and ND groups in both SRT and SDS. However, for both groups, the means were within normal limits. This finding can be explained because the frequencies that most contribute to the detection and discrimination of speech signals, which are between 500 and 2,000 Hz, did not show hearing threshold impairment in either group. Miller et al. 12 found similar results for speech tests between groups with and without diabetes and suggested that more sensitive speech tests (e.g., filtered speech tests) should be used in order to reveal the more subtle changes that occur within the auditory pathway.

Lastly, we conducted another comparison (Figure 1), excluding participants with a history of noise exposure, and found that mean hearing thresholds were not significantly different between the D and ND groups after individuals with history of noise exposure were removed from the analysis. This result indicates that hearing thresholds were not significantly affected by noise exposure in our study population. Similar findings were reported by Horikawa et al. 9.

Some studies have reported a positive association between the duration of diabetes and HL 25,26. However, the study populations and methods used in previous studies differ considerably from those in this study, which may explain the conflicting results. Sunkum and Pingile 25 included subjects with type 1 and 2 diabetes (5–55 years old), and the duration of disease ranged from ≈1–10 years. Conversely, Ozel et al. 26 included only subjects with type 1 diabetes (23–60 years old), but the authors did not report fasting blood glucose levels; in addition, most participants were women (>65%), and the duration of disease ranged from ≈3–13 years.

Other studies reported no association between the duration of diabetes and progression of HL 6,25. The meta-analysis by Akinpelu et al. 6 found no significant relationship between SNHL and the duration of diabetes in a pooled analysis of the data. The authors observed that only one study showed a positive association, whereas four failed to find any significant relationship. Similarly, Agarwal et al. 24 found no association between the duration of diabetes and progression of HL in a study that included subjects with type 1 and 2 diabetes (18–50 years old) and a time from diagnosis ranging from “recently diagnosed” to >5 years. The differences in study populations and methods across studies may explain the controversial findings, and thus the association between HL and duration of diabetes remains unclear 22. Cohort studies that examine hearing thresholds among people with diabetes over time may provide a more clear understanding of this relationship.

Another issue regarding the effect of diabetes on HL that may have affected the results of previous studies is that many studies have relied on self-reporting by participants 6,9,23, which may result in biased underestimation of diabetes cases 30. In our study, blood parameters including fasting serum glucose, HbA1c levels, and 2-h oral glucose tolerance, which are the main criteria for the diagnosis of diabetes along with clinical symptoms, were also measured and further support our findings.

In a review by Hong et al. 30, the authors discussed some theories about the physiological mechanisms that might explain HL in diabetes, such as microangiopathy, advanced glycation end products, and reactive oxygen species, among others.

Microangiopathy resulting from hyperglycemia may affect the cochlea, which is highly vascularized 7,12,30. Moreover, vascular changes to nervous tissue could cause ischemia, resulting in the loss of nerve fibers and demyelination 7.

Kakarlapudi et al. 22 found that increasing serum creatinine levels correlated with worse hearing in a subject with diabetes who had SNHL likely as a result of microangiopathy in the inner ear. Similarly, Agarwal et al. 24 reported that subjects with good glycemic control had significantly better hearing thresholds than those with poor glycemic control (HbA1c >58 mmol/mol). Conversely, Asma et al. 17 found no significant relationship between HL and glycemic control.

The lack of an association between diabetes and HL in our study may be because our study population was relatively young and most subjects had adequate glycemic control (78.4% of participants in D group) at ELSA-Brasil baseline. Thus, we assume that these participants did not have advanced microangiopathy, and their cochlear tissues were not directly affected by diabetes due to good control of the condition. This hypothesis could explain the lack of difference in hearing thresholds between the D and ND groups after adjusting for age, gender, and hypertension.

The adequate glycemic control in our study population may be related to the nature of ELSA-Brasil, as cohort volunteers are potentially more health-conscious than the general population. Thus, this fact should be considered when comparing our results to those from other cross-sectional analyses.

This study found no association between diabetes and worse hearing thresholds after adjusting for age, gender, and presence of hypertension. It should be noted that the diagnosis of diabetes was based on objective measures, and we also included confounding variables such as age, gender, presence of hypertension, noise exposure, and duration of diabetes, which have not been examined simultaneously in all studies on the effect of diabetes on SNHL. However, the mean age of participants and the duration of diabetes were relatively low in our study population, and glycemic control was adequate, which may have affected our findings.

Hearing thresholds and speech test results were significantly worse in D than in ND participants. However, after adjusting for age, gender, and the presence of hypertension, no significant differences were found between the groups. In addition, hearing thresholds were not affected by noise exposure in D or ND participants. Together, these findings revealed no association between the duration of diabetes and worse hearing thresholds after adjusting for age, gender, and hypertension.

## AUTHOR CONTRIBUTIONS

Samelli AG conceived and designed the study, was responsible for acquisition, analysis and interpretation of data, drafted and critically revised the manuscript for important intellectual content and approved the manuscript final version to be published. Santos IS participated in the analysis and interpretation of data, critically revised the manuscript for important intellectual content and approved the manuscript final version to be published. Moreira RR, Rabelo CM and Rolim LP participated in the acquisition of data, critically revised the manuscript for important intellectual content and approved the manuscript final version to be published. Bensenõr IJ revised the article critically for important intellectual content and approved the final version of the manuscript to be published. Lotufo PA critically revised the manuscript for important intellectual content and approved the final version of the manuscript to be published.

## Figures and Tables

**Table 1:** Baseline characteristics of study participants.

	Without diabetes (ND) N = 710	With diabetes (D) N = 191	*p*
Age (years) (Mean ± SD)	51.2 ± 8.9	57.4 ± 9.0	**<0.001**
Women sex (%)	389 (54.8)	87 (45.5)	**0.029**
Glucose (mmol/l)	5.76 ± 0.47	7.97 ± 2.76	**<0.001**
HbA1c (%) (mmol/mol)	5.3 ± 0.6 (34 ± 6.6)	6.5 ± 1.3 (48 ± 14.3)	**<0.001**
Hypertension (%)	185 (26.1)	112 (58.9)	**<0.001**
Systolic BP (mmHg)(Mean ± SD)	118.4 ± 14.9	128.1 ± 19.3	**<0.001**
Diastolic BP (mmHg)(Mean ± SD)	74.8 ± 10.1	79.4 ± 11.1	**<0.001**
Dyslipidemia (%)	391 (55.1)	118 (61.8)	0.115
Total cholesterol (mmol/l) (Mean ± SD)	5.48 ± 0.98	5.41 ± 1.09	0.221
LDL cholesterol (mmol/l) (Mean ± SD)	3.38 ± 0.84	3.30 ± 0.96	0.107
HDL cholesterol (mmol/l) (Mean ± SD)	1.45 ± 0.35	1.31 ± 0.29	**<0.001**
Triglycerides (mmol/l) (Mean ± SD)	1.47 ± 1.03	1.82 ± 1.15	**<0.001**
Creatinine (mg/dl) (Mean ± SD)	0.93 ± 0.22	0.99 ± 0.24	**0.001**
250 Hz (dBHL)(Mean ± SD)	12.6 ± 7.8	14.6 ± 8.9	**0.002**
500 Hz (dBHL)(Mean ± SD)	11.5 ± 8.1	13.9 ± 9.1	**<0.001**
1000 Hz(dBHL)(Mean ± SD)	11.6 ± 9.0	13.8 ± 10.1	**<0.001**
2000 Hz(dBHL)(Mean ± SD)	12.6 ± 11.1	16.2 ± 12.2	**<0.001**
3000 Hz(dBHL)(Mean ± SD)	15.3 ± 13.5	20.3 ± 14.9	**<0.001**
4000 Hz(dBHL)(Mean ± SD)	18.8 ± 15.5	25.1 ± 16.8	**<0.001**
6000 Hz(dBHL)(Mean ± SD)	25.1 ± 17.4	32.2 ± 18.2	**<0.001**
8000 Hz(dBHL)(Mean ± SD)	24.1 ± 19.6	31.7 ± 20.6	**<0.001**
SRT (dBHL)(Mean ± SD)	13.6 ± 8.0	16.2 ± 9.1	**<0.001**
SDS (%) (Mean ± SD)	96.2 ± 4.8	94.9 ± 6.3	**0.001**

SD = standard deviation; BP = blood pressure

**Table 2:** Individuals (n) with hearing impairment in each range frequencies (low-middle or high) for both groups (N and ND), odds-ratio and p-value in crude and adjusted model.

	Low-middle range frequencies. n (%) hearing impairment	High range frequencies. n (%) hearing impairment
Without diabetes (ND) N = 710	40 (5.6%)	187 (26.3%)
With diabetes (D) N = 191	20 (10.5%)	88 (46.3%)
Crude model (OR; 95% CI)	1.96 (1.12 – 3.44)	2.39 (1.72 – 3.32)
p-value	0.019	<0.001
Adjusted model (OR; 95% CI)	1.03 (0.56 – 1.92)	1.18 (0.78 – 1.78)
p-value	0.915	0.446

Adjusted model is adjusted for age, sex and hypertension diagnosis. OR: Odds ratio. 95% CI: 95% confidence interval.

**Table 3:** Beta-coefficients for the association between mean audiometric measurements and diabetes mellitus in crude and adjusted models.

	Crude	Adjusted for age	Full adjusted
250 Hz	1.99 (0.71 to 3.27; *p*=0.002)	0.66 (-0.64 to 1.95; *p*=0.319)	0.59 (-0.73 to 1.92; *p*=0.378)
500 Hz	2.39 (1.06 to 3.71; *p*<0.001)	0.84 (-0.48 to 2.17; *p*=0.213)	0.69 (-0.68 to 2.05; *p*=0.323)
1000 Hz	2.26 (0.77 to 3.74; *p*=0.003)	0.20 (-1.26 to 1.67; *p*=0.786)	0.18 (-1.33 to 1.69; *p*=0.815)
2000 Hz	3.63 (1.82 to 5.44; *p*<0.001)	0.34 (-1.38 to 2.06; *p*=0.696)	0.22 (-1.54 to 1.98; *p*=0.807)
3000 Hz	5.08 (2.88 to 7.28; *p*<0.001)	0.96 (-1.11 to 3.03; *p*=0.363)	0.69 (-1.38 to 2.77; *p*=0.513)
4000 Hz	6.23 (3.71 to 8.75; *p*<0.001)	1.39 (-0.96 to 3.74; *p*=0.247)	0.93 (-1.39 to 3.25; *p*=0.433)
6000 Hz	7.10 (4.30to 9.91; *p*<0.001)	1.18 (-1.38 to 3.74; *p*=0.366)	1.08 (-1.51 to 3.66; *p*=0.415)
8000 Hz	7.58 (4.41 to 10.75; *p*<0.001)	0.07 (-2.70to 2.84; *p*=0.960)	0.13 (-2.68 to 2.95; *p*=0.926)
SRT	2.55 (1.22 to 3.87; *p*<0.001)	0.25 (-1.01 to 1.51; *p*=0.701)	0.08 (-1.21 to 1.38; *p*=0.900)
SDS	-1.28 (-2.11 to -0.45; *p*=0.003)	-0.37 (-1.20 to 0.47; *p*=0.390)	-0.31 (-1.16 to 0.54; *p*=0.476)

Full adjusted models are adjusted for age, sex and hypertension diagnosis.
